# Applications of conductive hydrogels in sports performance monitoring

**DOI:** 10.1039/d6na00545d

**Published:** 2026-08-18

**Authors:** Jiahang Liu, Xiuya Zhang, Shijun Liu

**Affiliations:** a Tianjin University of Sport Tianjin 301617 China ljh@tjus.edu.cn; b Tianjin University of Foreign Studies Tianjin 300202 China

## Abstract

Conductive hydrogels, as functional materials with softness, electrical conductivity, and biocompatibility, have attracted increasing attention in the field of sports performance monitoring in recent years. Compared with traditional rigid sensors, conductive hydrogels can better conform to the skin and soft tissues, enabling stable and continuous signal acquisition during high-intensity exercise and in complex environments. This review comprehensively summarizes recent advances in the application of conductive hydrogels for sports monitoring, with a particular focus on material design strategies, conductive mechanisms, and key performance characteristics. Furthermore, typical application scenarios are discussed, including joint posture tracking, impact force measurement, electromyographic signal acquisition, sweat analysis, and feedback during rehabilitation training. The major challenges currently facing this field are also analyzed, such as insufficient durability, performance degradation in high-humidity environments, technical bottlenecks in multimodal synchronous signal acquisition, complex fabrication processes, and the lack of standardized evaluation systems. Finally, potential future development directions are proposed, including intelligence, multimodal integration, industrial translation, and green sustainable development. Conductive hydrogels are expected to become core materials for next-generation wearable sports monitoring devices, providing strong support for scientific training, injury prevention, and personalized rehabilitation.

## Abstract 2

Schematic of multifunctional conductive hydrogels integrating ionic/electronic conduction, self-healing and pH-responsive properties for smart sports monitoring, biomechanical sensing, physiological data acquisition and rehabilitation feedback.

## Graphical abstract

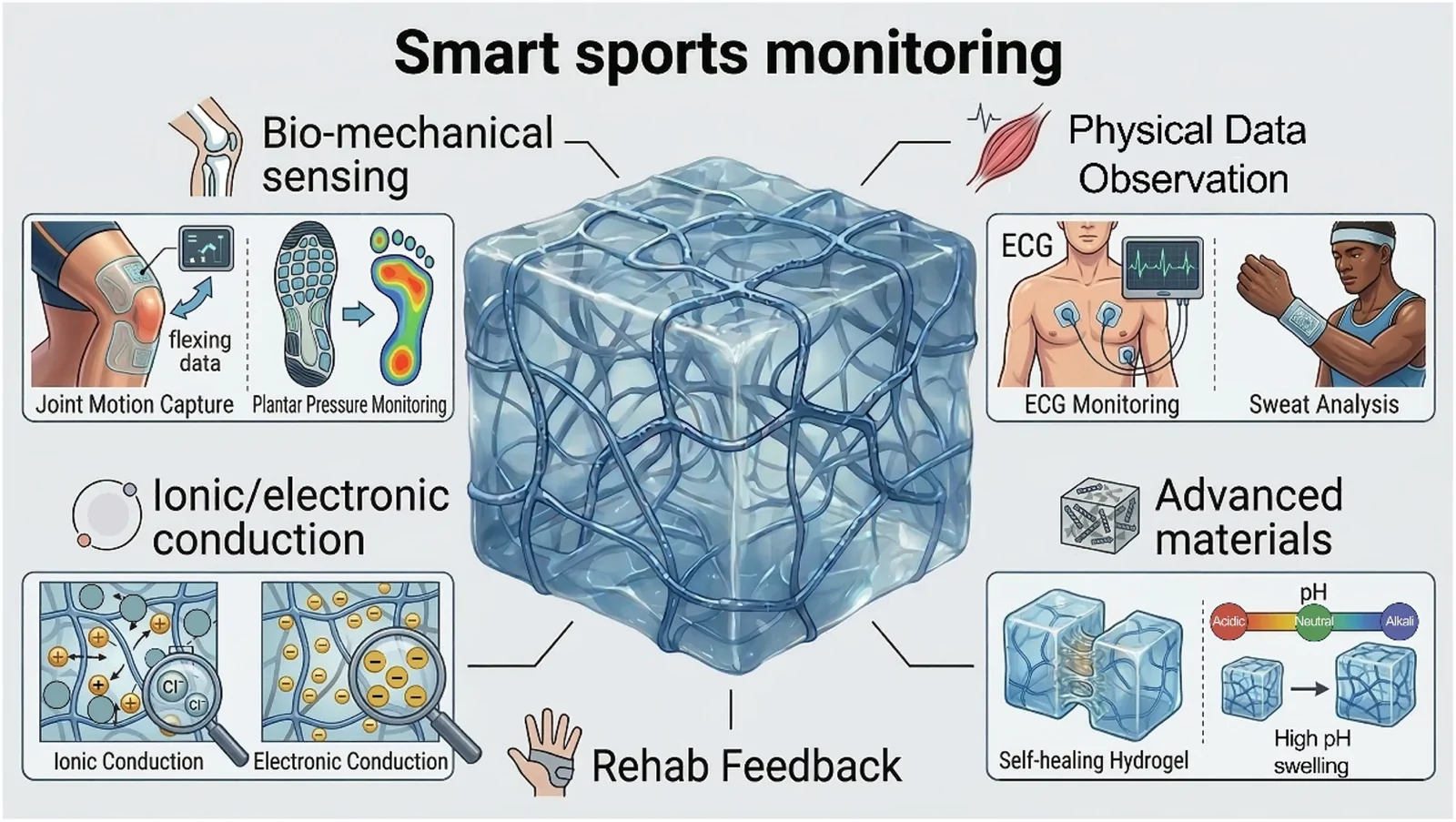

## 1. Introduction

As a central nexus integrating modern sports science, human performance science, and rehabilitation medicine, sports performance monitoring aims to decode the biomechanical and physiological-metabolic evolution of the human body under highly dynamic conditions through high-precision fundamental data acquisition.^1–3^ At the highest level of competitive sports, millisecond-scale deviations in movement execution or subtle fluctuations in physiological indicators may determine the outcome of competition and may even trigger catastrophic injuries. Accordingly, modern training systems are undergoing a paradigm shift from experience-driven subjective judgment toward multidimensional, data-driven precision quantification.^4–6^ Through real-time tracking of joint dynamics, landing impact forces, and surface electromyography, monitoring platforms not only provide optimal load matching for athletes seeking to push physiological limits, but also play an irreplaceable early-warning role in preventing severe acute injuries such as anterior cruciate ligament (ACL) rupture.^7–13^ Meanwhile, in the fields of postoperative clinical rehabilitation and chronic disease intervention for the general population, continuous and imperceptible physiological tracking is reshaping the boundaries of personalized medicine and providing critical digital evidence for the formulation of precise rehabilitation prescriptions.^14–19^

Although inertial measurement units, three-dimensional force platforms, and conventional medical Ag/AgCl electrodes have made historical contributions to the quantification of sports performance, their fundamental limitations have become increasingly prominent when confronted with the harsh requirements of real-world sports scenarios, including extremely high dynamics, intense physical contact, and complex environmental exposure.^20–24^ The first limitation lies in the irreconcilable “rigid-soft mismatch” and signal vulnerability. Traditional rigid silicon-based or metallic sensors cannot achieve conformal attachment to human skin, which is soft, highly elastic, and topographically complex.^25–27^ During intense sweating and large-deformation movements, such physical interfacial mismatch can easily induce severe interfacial slippage and motion artifacts, leading not only to device detachment and wearing discomfort, but also to the instantaneous masking of essential bioelectrical signals by massive mechanical noise.^28,29^ The second limitation concerns spatiotemporal constraints. High-precision optical motion capture systems and force platforms are highly dependent on expensive fixed laboratory settings, resulting in severe “laboratory fragmentation” of training data and preventing generalization to outdoor real-world competitive environments or long-term home-based rehabilitation scenarios.^30^

To bridge these technological gaps, conductive hydrogels, which combine tissue-level softness with excellent conductive networks, have emerged and rapidly become revolutionary materials reshaping the fundamental logic of wearable sports monitoring.^31–34^ As water-rich three-dimensional polymer networks, conductive hydrogels exhibit intrinsic advantages that are unmatched by conventional rigid electronics. First, they possess biomimetic tissue compliance and superior interfacial adhesion. Their tunable viscoelasticity closely matches that of skin, enabling seamless attachment during high-strain movements such as deep squatting and sprinting, thereby fundamentally alleviating dynamic micro-slippage.^35,36^ Second, conductive hydrogels enable multidimensional ionic–electronic conduction and high-fidelity signal acquisition with strong noise resistance. They can effectively mimic the ionic transport mechanism of natural neural conduction in the human body, allowing them to maintain low skin-electrode impedance and a stable signal-to-noise ratio (SNR) even in highly noisy environments with extensive sweat exposure.^37–39^ More importantly, through targeted molecular engineering, advanced conductive hydrogels have been endowed with additional functions such as ultrafast self-healing, wide-temperature-range anti-freezing capability, anti-dehydration properties, and biodegradability.^40–42^ These characteristics not only substantially extend the service life of materials in harsh environments such as ice-snow and underwater scenarios, but also align well with the global sustainable development strategy for green wearable electronics.^43^

With the rapid convergence of flexible electronics, the Internet of Things (IoT), and generative artificial intelligence (AI), evolving toward multimodal platforms with emerging capabilities in self-powering and edge computing, although fully integrated intelligent decision-making systems remain at an early stage of development. Given that this field is currently at a historical intersection of technological breakthroughs and industrial translation, a systematic review and strategic outlook are particularly urgent. Therefore, this review provides a panoramic analysis of the latest advances in conductive hydrogels for sports monitoring. Section 2 deeply dissects the fundamental conductive mechanisms of conductive hydrogels and state-of-the-art microscopic network design strategies, such as double-network architectures and topological anti-freezing designs. Section 3 systematically summarizes the core multimodal monitoring indicators related to biomechanics and physiological metabolism, as well as their corresponding material requirements. Section 4 focuses on disruptive application scenarios in competitive performance monitoring, injury prevention, and intelligent rehabilitation. Section 5 directly addresses the practical “valley of death” that hinders the translation of this technology from the laboratory to real-world sports settings, including material fatigue, multimodal spatiotemporal desynchronization, and barriers to commercial-scale manufacturing. Finally, Sections 6 and 7, from the broader perspectives of the Internet of Bodies and digital twins, propose a five-dimensional strategic roadmap for the development of next-generation intelligent hydrogel-based sports monitoring systems.

While several recent reviews have comprehensively covered the general design and applications of conductive hydrogels in wearable sensors and flexible electronics, a focused and critical analysis dedicated to the highly dynamic, high-sweat, and impact-intensive environment of sports performance monitoring remains limited. Existing reviews often emphasize material synthesis or broad biomedical sensing, yet pay relatively less attention to the specific biomechanical-physiological indicator systems required in competitive sports, the harsh service conditions (repeated large strain, continuous sweating, extreme temperatures), and the practical barriers to commercialization (Table 1) This review therefore aims to bridge this gap by systematically linking advanced hydrogel architectures to sports-specific monitoring requirements, critically examining translation challenges, and outlining a multi-dimensional roadmap toward next-generation intelligent sports monitoring platforms.

**Table 1:** Comparison of the present review with representative recent reviews on conductive hydrogels and wearable sensors (2025–2026)

Review (year)	Main focus	Sports-specific biomechanical & physiological indicators	Critical discussion of translation/commercialization barriers	Emphasis on AI & data fusion	Distinct contribution of the present review	Ref.
Gels, 2025, 11, 589	Conductive hydrogel flexible sensors for sports: Mechanisms & modification strategies	Moderate (motion, electrophysiological, electrochemical)	Limited	Limited	—	1
Polymer testing, 2026	Hydrogel wearable devices for sports monitoring & rehabilitation	Strong on applications in training, injury prevention, recovery	Moderate	Limited	—	3
Analyst, 2026/related early work	MXene-conductive hydrogels for wearable electrochemical sensors	Mainly electrochemical/sweat-related	Limited	Limited	—	31
Present review	Conductive hydrogels specifically for sports performance monitoring	Systematic classification of joint, impact, sEMG, sweat, fatigue & injury-risk indicators	In-depth analysis of durability, manufacturing, standardization, and “valley of death”	Dedicated discussion of AI and multimodal data-fusion strategies	Sports-scenario-driven material design + critical translation roadmap	—

## 2. Conductive hydrogel material design and performance

### 2.1 Basic performance requirements

As core functional materials for sports performance monitoring platforms, conductive hydrogels must be designed not only to meet the fundamental requirements of biomedicine and flexible electronics, but also to adapt to the complex and variable mechanical environments and high-dynamic signal acquisition demands encountered during sports training and competition (Fig. 1).^44,45^ Compared with traditional rigid electrodes and flexible thin-film sensors, conductive hydrogels are required to exhibit excellent comprehensive performance in three key dimensions: mechanical properties, electrical properties, and long-term wearing comfort. These characteristics are essential for ensuring highly sensitive and stable motion data acquisition during real-world sports activities.

**Fig. 1:**
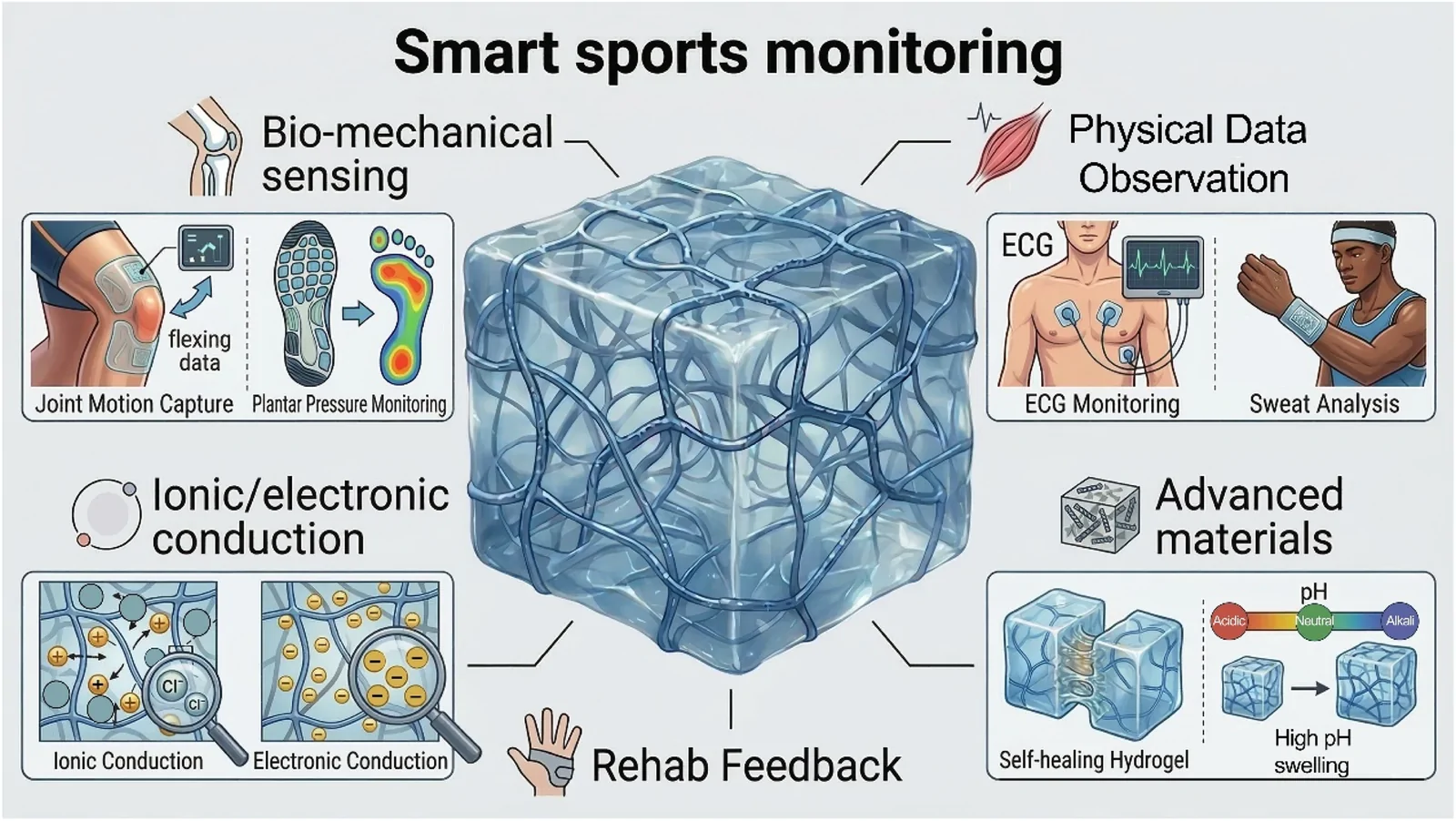
Schematic diagram of multifunctional conductive hydrogels featuring integrated ionic/electronic conduction and advanced material properties (*e.g.*, self-healing and pH-responsiveness) for smart sports monitoring, bio-mechanical sensing, physiological data observation, and rehabilitation feedback.

#### 2.1.1 Mechanical properties

At the mechanical level, sensors are required to withstand multidirectional tensile, compressive, and shear stresses. During high-intensity activities such as jumping and sprinting, conductive hydrogels must possess excellent softness, stretchability, and fatigue resistance to achieve high conformability with skin deformation.^46^ Ideally, their elastic modulus should be comparable to that of human skin to avoid local stress concentration. Considering that skin deformation during exercise can reach 50–70%, hydrogels must maintain structural integrity even under large strains.^47^ In addition, the cyclic stresses generated during long-term training require materials with outstanding fatigue resistance or self-healing capability to prevent structural rupture and signal interruption. Therefore, high flexibility and high toughness are primary design objectives for conductive hydrogels used in sports performance monitoring.^48^

#### 2.1.2 Electrical properties

Electrical performance is central to the function of conductive hydrogels. Sports performance monitoring often involves the acquisition of weak physiological signals, such as electromyographic (EMG) signals, which imposes stringent requirements on conductivity and signal stability.^49^ High conductivity ensures sensitive signal transmission under low operating voltages, while hydrogels must also maintain low-noise output during dynamic movements and effectively resist interference caused by sweat, friction, and electromagnetic disturbances. More importantly, hydrogels need to establish a stable, low-impedance contact interface with the skin, thereby substantially reducing contact artifacts and improving signal accuracy for high-precision monitoring.^50^

#### 2.1.3 Long-term wearing comfort

In addition, long-term wearing comfort should not be overlooked. Monitoring sessions lasting several hours require materials with good breathability to prevent sweat accumulation, local moisture retention, bacterial growth, and skin irritation or allergic responses.^51^ During vigorous exercise and heavy sweating, hydrogels must also maintain strong adhesion to prevent slippage or detachment and ensure signal continuity. Moreover, self-healing properties allow damaged materials to recover rapidly, significantly extending device lifespan and reducing maintenance costs.^52^ Together, these characteristics ensure the excellent adaptability of conductive hydrogels in complex application scenarios.

Based on the above performance requirements, this review argues that the future design of conductive hydrogels should no longer blindly pursue a single physical extreme, such as a static stretchability exceeding 1000%. Instead, it should shift toward balanced comprehensive performance under dynamic service conditions. Mechanically, research should focus on dynamic fatigue resistance and low energy hysteresis to cope with high-frequency gait cycles. Electrically, it is necessary to develop microstructured interfaces with sweat-swelling resistance or sweat-guiding capability to maintain a stable signal-to-noise ratio (SNR) during intense sweating. In terms of wearing experience, the key challenge lies in resolving the trade-off between firm attachment and on-demand painless removal. Only by achieving the organic integration of mechanical robustness, electrical stability, and wearing comfort can conductive hydrogels bridge the gap between laboratory prototypes and commercial wearable devices.

### 2.2 Classification of conductive mechanisms

The conductivity of conductive hydrogels mainly originates from internal charge-transport networks. According to their conduction mechanisms, conductive hydrogels can be classified into three major categories: ionic, electronic, and hybrid conductive hydrogels.^53^ As shown in Table 2 these three mechanisms differ substantially in typical components, conductive characteristics, and mechanical performance, which determines their specific application scenarios in sports performance monitoring, such as long-term sweat monitoring, high-frequency EMG acquisition, or complex rehabilitation assessment. Combined with the structural design and synthesis strategies illustrated in Fig. 2, the intrinsic advantages of each mechanism can be further amplified to meet diverse signal acquisition requirements.

**Table 2:** Comparison of design strategies and performance characteristics of different conductive hydrogels

Type	Conductive mechanism	Typical components	Typical conductivity	Stretchability	Key advantages	Key limitations	Preferred scenarios	Ref.
Ionic conductive hydrogels	Ionic conduction	NaCl, KCl	10^−3^-several S m^−1^	Often >500%	Biocompatibility, low cost, good skin conformity	Dehydration & ion leakage; possible high-frequency hysteresis	Long-term wear, sweat monitoring	55 and 56
Electronic conductive hydrogels	Electronic conduction	CNTs, PEDOT : PSS	1–10^2^ S m^−1^ (percolated)	100–600%	High sensitivity, strong anti-interference, better high-frequency response	Nanofiller toxicity; conductivity-flexibility trade-off	High-frequency EMG, intense monitoring	57–59
Hybrid conductive hydrogels	Synergistic electronic–ionic conduction	Salt ions + CNTs or conductive polymers	High & stable	300–1500%	High conductivity + stability; improved environmental tolerance	Interface mismatch; complex design	Multimodal sports monitoring & rehabilitation	60–62

**Fig. 2:**
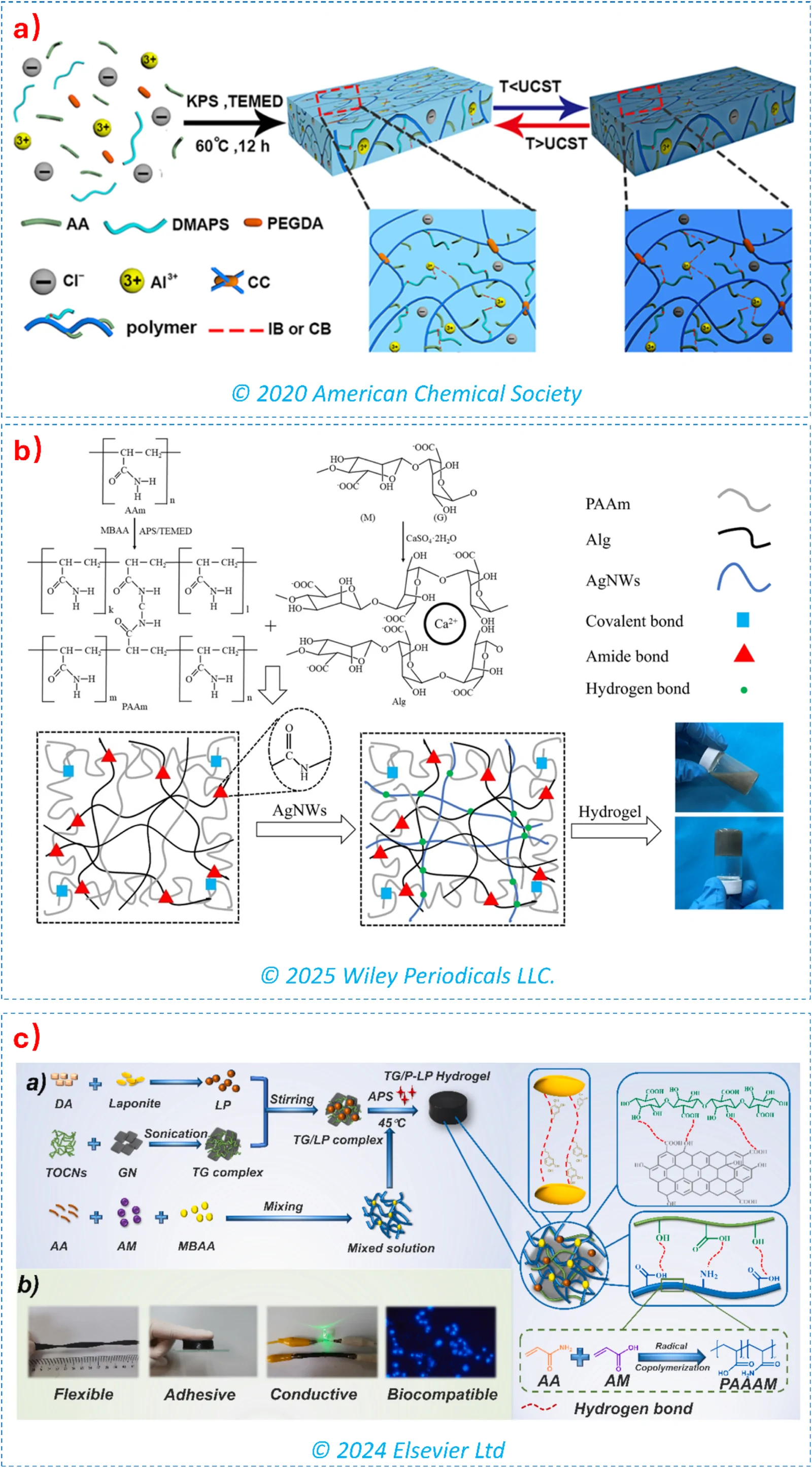
Advanced structural design and conductive mechanisms of multifunctional hydrogels: (a) Schematic illustration of the synthesis of dual cross-linked, temperature-responsive (UCST) ionic conductive hydrogels tailored for steady electrocardiogram (ECG) monitoring. (b) The formation mechanism of AgNWs-integrated electronic conductive hydrogels, highlighting synergistic dynamic interactions (covalent, amide, and hydrogen bonds) for exceptional mechanical toughness and conductivity. (c) The fabrication process and comprehensive properties (*e.g.*, self-adhesion, UV shielding, antioxidant activity, and biocompatibility) of lignin/polyphenol-based composite conductive hydrogels for multi-modal wearable bioelectronics.

#### 2.2.1 Ionic conductive hydrogels

Ionic conductive hydrogels rely on the migration of hydrated ions derived from salts or simulated body fluids to transport charges.^54^ This conduction mechanism is naturally compatible with physiological bioelectrical signals and exhibits excellent biocompatibility. As shown in Fig. 2a, by introducing zwitterionic monomers and metal ions (Al^3+^), an ionic conductive network with a dual cross-linked structure can be successfully constructed. This sophisticated design not only endows the hydrogel with a distinctive UCST phase-transition behavior, enabling intelligent thermoresponsiveness, but also allows it to maintain extremely low impedance fluctuations under complex dynamic deformation. As a result, stable and continuous ECG and multimodal physiological signal monitoring can be achieved.^55^ However, the conductive stability of ionic hydrogels is strongly constrained by environmental dehydration and ion dissipation, which may lead to performance degradation during long-term service. In addition, owing to the physical limitation of ion migration rates, ionic conductive hydrogels may exhibit response hysteresis during high-frequency dynamic signal acquisition.^56^

#### 2.2.2 Electronic conductive hydrogels

Electronic conductive hydrogels achieve efficient charge transport by introducing nanoscale conductive materials or conductive polymers, with electrons or holes serving as the primary charge carriers.^57^ As illustrated by the synthesis mechanism in Fig. 2b, one-dimensional silver nanowires (AgNWs) can be uniformly dispersed within a polyacrylamide (PAAm) and sodium alginate (Alg) matrix. Covalent bonds, amide bonds, and hydrogen bonds between rigid conductive components and polymer chains together construct a tough and synergistic network. Such multiple physical–chemical cross-linking not only effectively alleviates stress concentration caused by rigid fillers, thereby ensuring high mechanical performance and biocompatibility, but also provides a continuous and unobstructed three-dimensional percolation pathway for electron transport.^57,58^ Compared with ionic conductive hydrogels, electronic conductive hydrogels generally exhibit higher absolute conductivity and stronger resistance to electromagnetic interference, enabling precise capture of high-frequency EMG signals. Nevertheless, their fabrication processes are relatively complex, Although electronic conductive hydrogels often exhibit superior absolute conductivity and high-frequency response, the incorporation of nanoscale conductive fillers such as silver nanowires (AgNWs), carbon nanotubes (CNTs), or graphene raises legitimate biocompatibility concerns. Toxicity is strongly dependent on filler concentration, surface chemistry, exposure duration, and the specific cell line tested. Several recent studies have reported excellent short-term cytocompatibility (cell viability often exceeding 90–95%) for AgNW- and PEDOT : PSS-based hydrogels at optimized low loadings. However, higher filler concentrations or potential long-term ion/particle leaching under continuous mechanical deformation and sweat exposure may still induce oxidative stress or inflammatory responses. Therefore, simply claiming “biocompatibility” without quantitative dose–response data is insufficient. Current mitigation strategies include surface passivation or polymer coating of nanofillers, minimizing the filler content needed to reach percolation, and increasingly replacing metallic or carbon nanomaterials with inherently more biocompatible conductive components such as PEDOT : PSS, lignin, or natural polyphenols. Rigorous long-term *in vitro* and *in vivo* evaluations under realistic sports-related conditions remain essential before clinical or consumer-level adoption,^57–59^ and excessive filler loading can compromise the intrinsic flexibility of the hydrogel matrix.

#### 2.2.3 Hybrid conductive hydrogels

Hybrid conductive hydrogels integrate both ionic and electronic conduction pathways and represent an important breakthrough in flexible sensing materials.^60^ As shown in Fig. 2c, composite systems constructed from lignin or natural polyphenols not only achieve a favorable balance between flexibility and high conductivity, but also impart excellent multifunctional properties to the material. The abundant polyphenolic groups and quinone structures in lignin, on the one hand, provide hydrogels with excellent self-adhesion through dynamic physical cross-linking; on the other hand, their inherent ultraviolet-shielding and antioxidant activities can effectively protect the gel from environmental photodegradation and sweat-induced oxidative erosion.^61^ At the microscopic level, ionic channels mimic the natural transmission of physiological signals and maintain network compliance, whereas electronic channels enable efficient interfacial charge transfer. The synergistic integration of these two pathways overcomes the limitations of a single conduction mechanism. Owing to their high signal stability and environmental tolerance, such materials are particularly suitable for high-intensity dynamic training and precise rehabilitation assessment, representing a core developmental direction for advanced wearable sensing materials.^62^

Overall, the above three mechanisms indicate that conductive hydrogels are evolving from single-mode conduction toward multidimensional synergy and multifunctional integration. Ionic conductive hydrogels need to overcome the bottleneck of dehydration-induced deactivation. Future designs should move beyond simple salt addition and instead introduce deep eutectic solvents (DESs) to achieve long-term water retention at the thermodynamic level. Electronic conductive hydrogels remain constrained by the trade-off between mechanical and electrical performance, which must be addressed through biomimetic interface engineering, such as monodisperse anchoring, to mitigate the adverse effects of rigid fillers on flexibility. Hybrid conductive hydrogels currently represent the most promising evolutionary solution. Their future development should focus not only on overcoming impedance mismatch at heterogeneous interfaces, but also on fully exploiting the potential of green multifunctional building blocks, such as lignin and natural polyphenols. The integration of biomimetic properties, including antioxidant activity, UV shielding, and self-healing, with scalable one-pot fabrication strategies will be a key breakthrough for advancing hybrid hydrogels toward all-weather, highly reliable, and intelligent sports monitoring.

Overall, a quantitative comparison reveals clear trade-offs. Ionic hydrogels typically offer superior softness (modulus in the low-kPa range) and biocompatibility but suffer from conductivity degradation upon dehydration. Electronic hydrogels can achieve higher absolute conductivity and better high-frequency response, yet often at the expense of mechanical compliance when rigid fillers are heavily loaded. Hybrid systems currently provide the most balanced performance, combining relatively high and stable conductivity with improved toughness and environmental tolerance, although challenges remain in optimizing the ionic–electronic interface and simplifying fabrication. These quantitative differences directly inform material selection for specific sports monitoring scenarios (*e.g.*, long-term sweat analysis *vs.* high-frequency EMG during intense training).

### 2.3 Typical structural design strategies

It should be noted that the high mechanical and functional performance reported for many conductive hydrogels does not originate from the individual polymer components alone. Pure polyacrylamide (PAAm) networks are relatively brittle, pure carboxymethyl cellulose (CMC) hydrogels typically exhibit limited mechanical strength, pure polyvinyl alcohol (PVA) hydrogels often suffer from limited long-term stability, and pure alginate systems face challenges in controlled cross-linking and mechanical robustness.^55–60^ The excellent stretchability, toughness, and sensing performance achieved in the cited studies therefore arise primarily from the carefully designed multi-network architectures, dynamic cross-linking strategies, and synergistic incorporation of conductive fillers.^61,62^ To meet the stringent requirements of complex sports scenarios, the structural design of conductive hydrogels has evolved from homogeneous networks toward multilevel synergistic architectures. As shown in Fig. 3, advanced designs can be mainly summarized into the following three typical strategies.

**Fig. 3:**
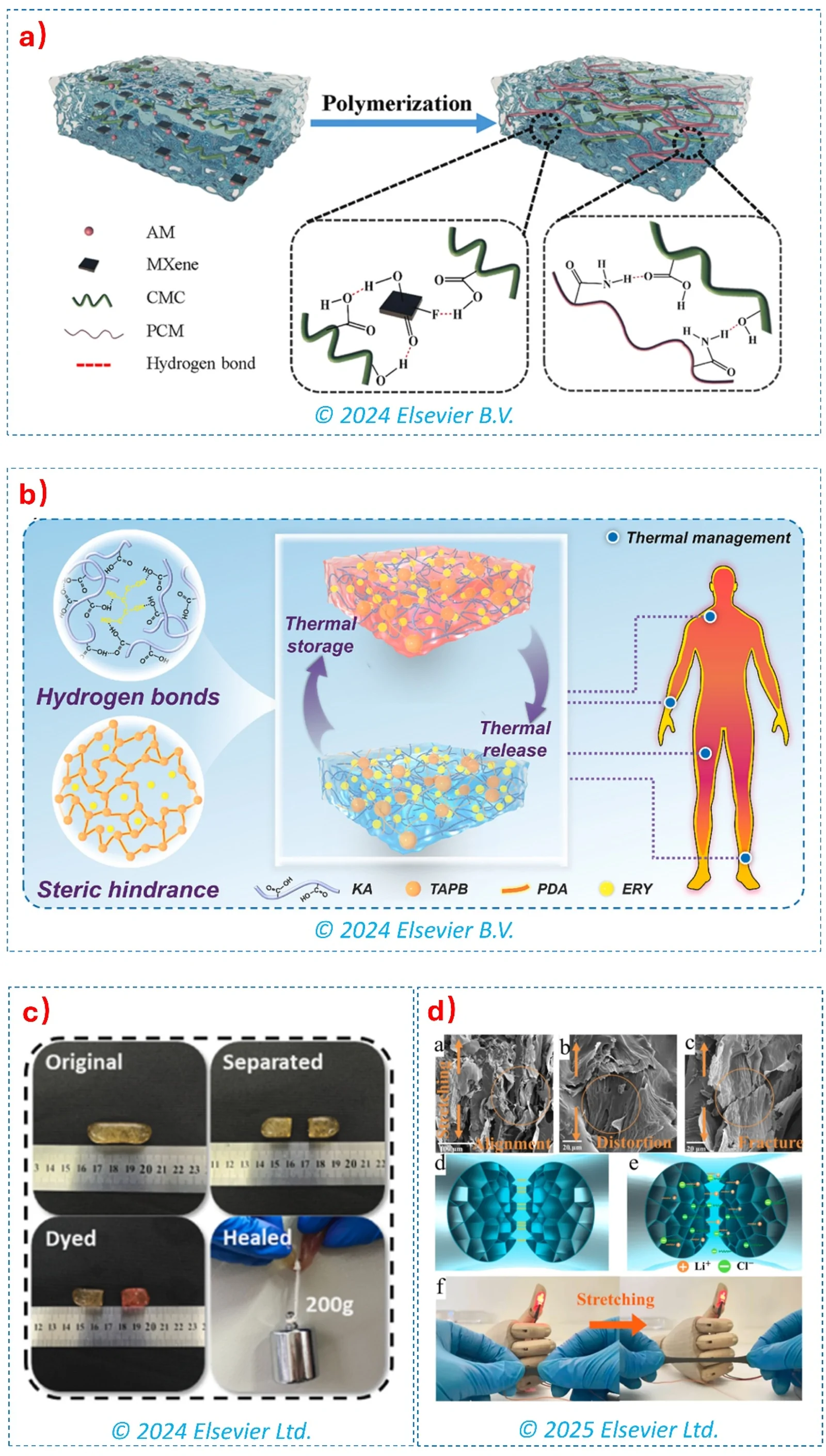
Advanced structural design strategies for multifunctional conductive hydrogels in wearable sports monitoring. (a) Schematic of a highly sensitive MXene-enhanced PAAm/CMC double-network (DN) hydrogel, highlighting the mechanical reinforcement and conductive pathways formed by tight hydrogen bonding. Reproduced with permission.^63^ Copyright 2024, Elsevier B.V. (b) Design of an erythritol-based spatiotemporal phase change film with a double-network structure, demonstrating solid-solid phase transition for effective wearable thermal management. Reproduced with permission.^64^ Copyright 2024, Elsevier B.V. (c) Macroscopic demonstration of a highly robust, “one-pot” synthesized collagen/PVA/tannin self-healing hydrogel, showing rapid macroscopic recovery of mechanical and electrical properties after severe damage. Reproduced with permission.^65^ Copyright 2024, Elsevier Ltd. (d) Microstructural mechanism and extreme environmental tolerance of a 3D graphene skeleton/PAAm/LiCl nanocomposite hydrogel, maintaining stable alignment and conductivity under immense strain and freezing conditions. Reproduced with permission.^66^ Copyright 2025, Elsevier Ltd.

#### 2.3.1 Double-network structural design coupled with thermal management

The double-network (DN) structure represents a classic paradigm for mechanical reinforcement. As shown in Fig. 3a, a strategy using PAAm as the main chain, CMC as a flexible subnetwork, and MXene to construct a hydrogen-bonding network endows the material with a wide detection range of 0–500% and high strength and toughness through the “sacrificial bond” mechanism, thereby effectively dissipating mechanical impacts during intense exercise.^63^ In addition, to address body-surface overheating induced by high-intensity competition, a flexible erythritol-based double-network spatiotemporal phase-change film, as shown in Fig. 3b, successfully overcomes the rigidity limitation of conventional phase-change materials. While maintaining high stretchability, it enables efficient thermal regulation through latent heat storage and release during phase transition, opening a new route for intelligent thermal management in wearable devices.^64^

#### 2.3.2 Self-healing structural design

Self-healing design endows materials with autonomous repair capability after damage by introducing reversible physical or chemical interactions. As shown in Fig. 3c, a collagen/PVA/tannic acid-based PBTC hydrogel prepared through a one-pot method can achieve a healing efficiency of over 90% within 90 s after macroscopic cutting, driven by dynamic hydrogen bonding. After healing, its stretchability remains above 1500%, and it can even lift a 200 g weight again.^65^ This ultrafast repair capability significantly improves the fatigue resistance of sensors during high-frequency monitoring and substantially extends device lifetime.

#### 2.3.3 Stretchable and environment-tolerant network design

For large-deformation and extreme outdoor monitoring, constructing a three-dimensional continuous conductive skeleton is a key strategy. As shown in Fig. 3d, a three-dimensional graphene skeleton/polyacrylamide (3DGS/PAM) system allows the skeleton to undergo conformal sliding during stretching and maintains highly stable electrical signals even under strains as high as 610% and after thousands of cycles. More importantly, the lyophilic ions within this microscopic network endow the hydrogel with excellent anti-freezing capability at −25 °C and long-term moisture retention, with a water content of 118 wt% after 10 days, making it highly suitable for real-time monitoring in extreme environments such as ice and snow sports.^66^

To facilitate a quantitative comparison of the performance of different conductive hydrogels under sports-relevant conditions, key metrics of representative systems are summarized in Table 3.

**Table 3:** Comparative summary of key performance metrics of representative conductive hydrogels discussed in this review

Hydrogel system	Conductivity	Max stretchability	Gauge factor/sensitivity	Electrical hysteresis	Self-healing	Environmental tolerance	Main application focus	Ref.
Dual cross-linked zwitterionic ionic hydrogel (UCST)	Extremely low impedance fluctuation under dynamic deformation	—	Stable ECG signal acquisition	—	—	Temperature-responsive	Steady multimodal physiological monitoring	55
AgNWs-integrated electronic conductive hydrogel	Continuous 3D percolation pathway	High toughness	High absolute conductivity	—	—	—	High-frequency EMG acquisition	57 and 58
Lignin/polyphenol-based hybrid conductive hydrogel	Balanced ionic–electronic conduction	Good flexibility + toughness	High signal stability	—	Self-adhesion *via* dynamic physical cross-linking	UV shielding + antioxidant	Multi-modal wearable bioelectronics	61
MXene-enhanced PAAm/CMC double-network	—	0–500% detection range	High sensitivity	—	—	—	Wide-range strain sensing for intense exercise	63
Collagen/PVA/tannic acid (PBTC) hydrogel	—	>1500% (after healing)	—	—	>90% healing efficiency within 90 s	—	Rapid self-healing motion sensors	65
3D graphene skeleton/PAAm/LiCl nanocomposite	Highly stable electrical signals under large strain	610%	Stable under cyclic loading	—	—	−25 °C anti-freezing; water content 118 wt% after 10 days	Extreme environment (ice/snow) monitoring	66

It should be noted that the numerical performance data presented in this review (*e.g.*, electrical hysteresis, stretchability, self-healing efficiency) are taken from the original literature. The specific measurement conditions—such as strain frequency, amplitude, temperature, humidity, and loading rate—often differ across studies and are not always fully reported. These parameters can significantly influence the apparent performance of hydrogels under dynamic conditions. Therefore, direct quantitative comparison should be interpreted with caution, and the establishment of standardized testing protocols is strongly recommended.

At the structural design level, conductive hydrogels are shifting from the pursuit of a single extreme property toward multiphysical-field coupling and all-weather environmental adaptability. Future design efforts should accelerate the transition toward multifunctional hierarchical architectures. Such architectures should not only resolve the fundamental trade-off between mechanical robustness and electrical conduction, as exemplified by three-dimensional graphene skeletons, but also deeply integrate environmentally responsive building blocks, such as phase-change micromaterials. Only by establishing a three-dimensional closed loop integrating mechanical toughness, high-frequency conductivity, and active thermal/water management can conductive hydrogels truly meet the requirements of reliable commercial sports monitoring.

The performance of conductive hydrogels varies markedly across different sports environments. Underwater or high-humidity conditions primarily challenge the network against excessive swelling and ion leakage; anti-swelling double-network designs have demonstrated retained high stretchability and stable sensing in aquatic or high-humidity settings.^35^ In high-temperature or intense-exercise scenarios that generate substantial body heat, the integration of phase-change materials (*e.g.*, erythritol-based double networks) enables active thermal regulation while preserving stretchability.^64^ For subzero environments typical of winter sports, the incorporation of lyophilic salts or ionic liquids has enabled reliable operation at −25 °C and even down to −40 °C, with maintained conductivity and water retention over extended periods.^66^ Despite these advances, most current systems are optimized for only one extreme condition. Hydrogels capable of simultaneously delivering high sensing fidelity under combined underwater, high-temperature, and subzero stresses remain scarce and represent a critical target for future all-weather sports monitoring platforms.

## 3. Monitoring requirements and key indicators for sports performance

With the rapid development of sports science and rehabilitation medicine, sports performance monitoring has shifted from traditional experience-driven assessment toward data-driven quantitative analysis.^63^ To meet the needs of practical applications, monitoring platforms are required not only to acquire multidimensional signals, but also to maintain high sensitivity and stability in complex dynamic scenarios,^67^ as illustrated in Fig. 4.

**Fig. 4:**
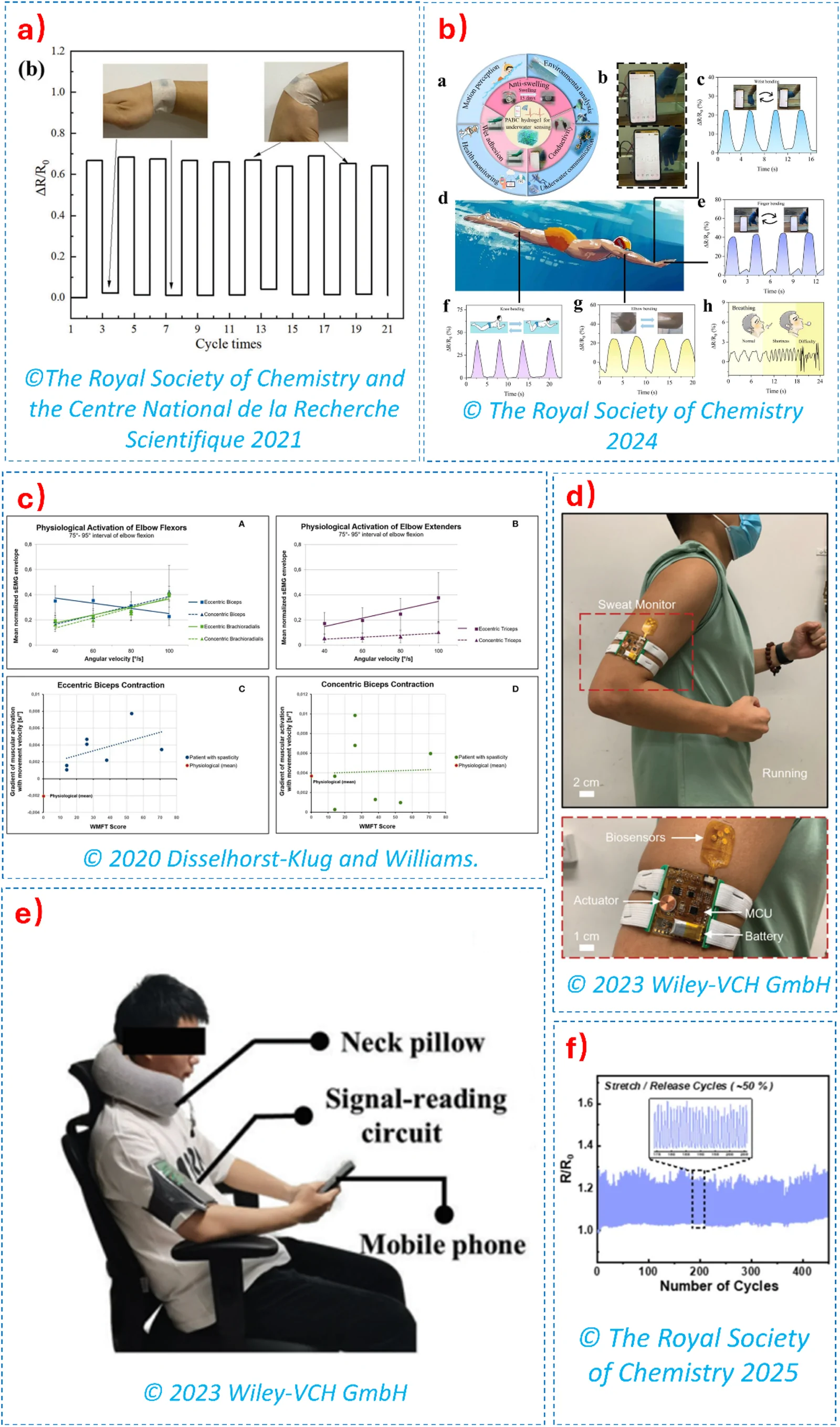
Comprehensive overview of critical monitoring indicators and advanced technical requirements for sports performance platforms. (a) Application of CNT-Br/PEDOT : PSS/PAAS three-network composite conductive hydrogels for highly sensitive human joint motion monitoring. Reproduced from ref. 69 with permission from the Royal Society of Chemistry,^69^ copyright 2021. (b) Deep learning-assisted (2D-CNN) underwater motion posture visualization enabled by anti-swelling dual-network hydrogels. Reproduced from ref. 70 with permission from the Royal Society of Chemistry,^70^ copyright 2024. (c) Integration of surface electromyography (sEMG) and biomechanical inputs for precise neuromuscular assessment. Reproduced from ref. 73 with permission from Frontiers,^73^ copyright 2020. (d) Intelligent soft sweat sensors equipped with microfluidics for simultaneous physiological monitoring and safety warnings. Reproduced from ref. 75 with permission from John Wiley and Sons,^75^ copyright 2023. (e) Wireless human motion detection utilizing highly sensitive LC-resonator wearable pressure sensing (LC-WPS) technology. Reproduced from ref. 82 with permission from John Wiley and Sons,^82^ copyright 2023. (f) Recyclable, highly stretchable alginate/PEDOT : PSS hydrogels exhibiting low electrical hysteresis for stable long-term wireless sensing. Reproduced from ref. 88 with permission from the Royal Society of Chemistry,^88^ copyright 2025.

### 3.1 Objectives of sports performance monitoring

The core objective of sports performance monitoring is to dynamically evaluate physiological and mechanical load, thereby providing scientific evidence for training optimization and injury prevention. In terms of training optimization, accurate data acquisition enables the assessment of the match between training load and athletic performance.^68^ As shown in Fig. 4a, a triple-network composite conductive hydrogel constructed from CNT-Br/PEDOT : PSS/PAAS exhibits a maximum conductivity of 10.35 mS cm^−1^. Owing to its excellent mechanical properties, it can capture joint movements with high fidelity, helping reduce the risk of injuries caused by overtraining.^69^ For early injury warning, as shown in Fig. 4b, an anti-swelling bacterial cellulose-based double-network hydrogel with stretchability exceeding 1304%, combined with a two-dimensional convolutional neural network (2D-CNN), can achieve a recognition accuracy of up to 99.37% for movements in complex environments such as underwater swimming, enabling early identification of potentially high-risk movement patterns.^70^ Such early-warning mechanisms can provide an important safeguard for athletes' careers.^71^ Meanwhile, in clinical rehabilitation, continuous data feedback can ensure movement standardization, prevent secondary injuries caused by compensatory movements, and accelerate recovery to pre-injury competitive levels.^72^

### 3.2 Classification of key monitoring indicators

To systematically construct a monitoring and evaluation framework, Table 4 summarizes key signal characteristics, technical requirements, and application scenarios across three dimensions: biomechanical, physiological, and integrated parameters. First, as shown in the upper part of Table 4 at the biomechanical and electromyographic levels, accurate acquisition of joint angles and impact forces is fundamental for preventing severe injuries such as ACL rupture. As illustrated in Fig. 4c, the correct interpretation of surface electromyographic (sEMG) signals during rehabilitation must incorporate biomechanical inputs such as muscle fiber length and contraction velocity. This is a core prerequisite for accurately evaluating muscle activation and coordination status.^73^ With these cross-dimensional mechanical indicators, coaches can identify abnormal movement patterns that may lead to fatigue accumulation.^74^

**Table 4:** Key indicators, technical requirements, and application scenarios for sports performance monitoring

Signal type	Specific indicator	Monitoring significance	Technical requirements	Typical application scenarios	Ref.
Biomechanical signals	Joint angle (°)	Evaluates the standardization of sports techniques and analyzes joint range of motion and athletic performance	High-precision angle sensing; real-time tracking; strong anti-interference capability	Running posture analysis; knee rehabilitation training; sports technique assessment	68 and 69
Biomechanical signals	Acceleration (m s^−2^)	Reflects changes in body or limb movement velocity and evaluates explosive power and coordination	High response speed; low-noise output	Sprint training; jump technique analysis; balance ability assessment	70 and 82
Biomechanical signals	Impact force/ground reaction force (N)	Analyzes landing impact and exercise load and predicts injury risk	Wide dynamic range; ability to withstand high-frequency impact	Landing movement assessment; ACL injury warning; optimization of footwear and sportswear design	71 and 72
Physiological signals	Electromyographic signal (EMG, mV)	Evaluates muscle activation level, coordination, and fatigue status	High sensitivity; low-voltage detection; low-impedance skin interface	Lower-limb muscle fatigue analysis; rehabilitation training feedback; neuromuscular control research	73 and 74
Physiological signals	Sweat ion concentration (Na^+^, K^+^, Cl^−^)	Monitors fluid balance and dehydration risk and evaluates metabolic status	Stable ion sensing; adaptability to high-humidity environments	Long-distance training; exercise monitoring in hot environments; hydration management	75
Integrated indicators	Fatigue index	Integrates EMG waveform, impact force, heart rate variability, and other indicators to evaluate overall fatigue level	Multimodal data fusion; real-time analysis	Training-cycle planning; overtraining warning	78–80

Second, as indicated in the middle section of Table 4 real-time feedback on body-fluid status and neural state is essential for physiological signal monitoring. As shown in Fig. 4d, intelligent soft sweat sensors collect sweat through hydrophilic microfluidic systems and can simultaneously detect Na^+^, glucose, pH, and skin impedance, thereby providing real-time warnings for dehydration risk and metabolic safety.^75^ When combined with EMG changes and psychological stress indicators such as skin conductance,^76,77^ these signals provide comprehensive physiological contextual information. Finally, the integrated indicators listed at the end of Table 4 suggest that modern intelligent monitoring is moving toward the construction of multidimensional models.^78^ By integrating the above multisource parameters to calculate fatigue and risk indices, monitoring systems can provide coaches with intuitive quantitative evidence for individualized training decisions, which also represents a central hub for the transition of flexible sensing platforms toward system-level applications.^79,80^

In relation to the stringent technical requirements listed in Table 4 conventional devices show clear limitations in wearing comfort and multimodal acquisition, whereas conductive hydrogels provide an ideal solution owing to their unique properties.^81^ The first requirement is high sensitivity combined with wireless transmission capability. Exercise-related physiological signals are weak and highly susceptible to interference. As shown in Fig. 4e, wireless pressure sensing technology based on an inductive-capacitive (LC) resonator, namely LC wireless pressure sensing (LC-WPS), achieves a resolution as high as 54 Pa using a single operating frequency of 13.56 MHz. This passive sensor can sensitively detect subtle deformations in regions such as the throat and wrist,^82^ while maintaining high response speed and stability under low-voltage conditions,^83^ thereby providing reliable data support for sudden physiological or mechanical changes.^84^

The second requirement is multimodal integrated sensing.^85^ Conductive hydrogels can synchronously acquire mechanical, physiological, and electrochemical signals, thereby reducing device redundancy and substantially improving data completeness and analytical efficiency.^86^ The final requirement is long-term comfortable wearing with extremely low hysteresis.^87^ During large-deformation movements, as shown in Fig. 4f, a recyclable alginate/PEDOT : PSS hydrogel cross-linked with CaCl_2_ not only achieves 138% stretchability, but also exhibits an extremely low electrical hysteresis of only 2.95% under 50% strain, maintaining high stability over 400 cycles.^88^ Together with the intrinsic self-healing properties of hydrogels,^89^ conductive hydrogels open a new pathway toward intelligent, real-time, and personalized sports monitoring.^90^

Overall, from the perspective of the fundamental requirements of sports monitoring, current monitoring systems are undergoing a paradigm transition from discrete single-point testing toward all-weather multimodal sensing integrated with deep learning. Future research and development of conductive hydrogels should advance simultaneously at both the hardware and algorithmic levels. At the material hardware level, it is necessary to overcome force–electrical–moisture interfacial instability under extreme sweating and intense stretching. Designs such as LC wireless pressure sensing (LC-WPS) and low-hysteresis conductive networks are critical for eliminating dynamic signal drift. At the algorithmic software level, lightweight edge-computing models, such as 2D-CNNs, should be deeply embedded to address the bottlenecks of low-latency synchronization and real-time warning for massive multisource data on wearable devices. Only through hardware–software synergy can conductive hydrogels truly establish a closed loop from raw physiological signals to intelligent decision-making in real-world sports scenarios.

### 3.3 Comparison with commercial sports-monitoring devices

Relative to conventional Ag/AgCl electrodes, which remain the clinical gold standard for sEMG,^20,29^ conductive hydrogel electrodes currently show a characteristic trade-off. Under static or low-motion conditions, Ag/AgCl electrodes typically deliver higher absolute signal-to-noise ratios and benefit from decades of standardization and clinical validation. However, during dynamic sports activities involving large skin deformation and heavy sweating, Ag/AgCl electrodes are prone to interfacial slippage, increased contact impedance, and motion artifacts that can severely degrade signal quality.^28,29^ In contrast, soft conductive hydrogels form a more conformal and stable skin interface, maintain lower impedance under strain, and exhibit improved artifact resistance in high-intensity movements.^37–39,50^ Nevertheless, most reported hydrogel EMG sensors still require further improvement in long-term operational stability, batch consistency, and quantitative benchmarking against clinical-grade Ag/AgCl systems before they can fully replace conventional electrodes in professional sports or medical settings.

Compared with commercial sports-monitoring devices, conductive hydrogel sensors currently show both clear advantages and remaining limitations. Conventional Ag/AgCl electrodes and inertial measurement units (IMUs) offer high signal fidelity and established standardization under controlled conditions, while optical motion-capture systems and force platforms remain the gold standard for precise kinematic and kinetic quantification in laboratory settings. However, these commercial solutions often suffer from rigid-soft mismatch, limited skin conformity during large-strain movements, susceptibility to motion artifacts and sweat interference, and restricted usability outside fixed laboratory environments. In contrast, conductive hydrogels provide tissue-like softness, superior interfacial adhesion under dynamic deformation, and the inherent ability to integrate mechanical, electromyographic, and biochemical sensing within a single soft platform. Nevertheless, hydrogel-based sensors still lag behind commercial devices in long-term operational stability, batch-to-batch reproducibility, and standardized performance evaluation protocols. Bridging this gap will be essential for their practical adoption in real-world sports monitoring.

To provide a clearer comparison with existing commercial technologies, Table 5 summarizes the key performance differences between conductive hydrogel sensors and representative commercial sports-monitoring devices (Ag/AgCl electrodes, inertial measurement units, and optical motion-capture/force platforms).^25–30^ Commercial systems currently offer higher absolute accuracy, longer service life, and better standardization under controlled conditions. In contrast, conductive hydrogels exhibit superior skin conformity, reduced motion artifacts during large-strain movements, better tolerance to sweat, and greater potential for multimodal integration in real-world sports environments.^29,37–39^ Bridging the remaining gaps in durability and standardization remains essential for practical adoption. The key differences discussed above are summarized in Table 5 for clearer comparison.

**Table 5:** Comparison of conductive hydrogel sensors with commercial sports-monitoring devices

Metric	Conductive hydrogel sensors	Ag/AgCl electrodes	IMUs	Optical motion capture/force platforms	Ref.
Skin conformity & wearability	Excellent	Moderate	Good	Poor (lab-only)	20 and 25–27
Motion artifact under large strain	Low	High	Low–moderate	Minimal	28 and 29
Sweat interference resistance	Moderate to good	Poor	Good	Not applicable	37–39
Multimodal capability	High	Limited	Limited	Limited	20
Outdoor usability	Emerging	Limited	Good	Poor	35 and 40–42
Service life & standardization	Currently limited	High	High	High	20 and 30

## 4. Applications of conductive hydrogel platforms in sports performance monitoring

Owing to their intrinsic flexibility and tunable physicochemical properties, conductive hydrogels have successfully evolved from single-mode mechanical sensors into multimodal, self-powered, and IoT-enabled systems, greatly promoting the development of sports monitoring toward real-time, boundaryless, and intelligent applications.^91^

### 4.1 Motion signal acquisition platforms

#### 4.1.1 Motion posture and all-weather joint monitoring

Accurate reconstruction of motion posture requires sensors to maintain extremely high signal fidelity under large deformation. As shown in Fig. 5a, a composite hydrogel based on PEDOT : PSS/PAAm-SA achieves a high gauge factor (GF) and an ultralow electrical hysteresis of only 1.52% within a working strain range of up to 500%. Integrated with a wireless communication module, this system can seamlessly and without delay reconstruct finger bending and complex limb movements.^92^ In addition, to overcome the limitations of conventional optical devices in outdoor environments, an all-weather ionic hydrogel incorporating hydrated ionic liquids, as shown in Fig. 5b, not only achieves a high transparency of 94.36%, but also broadens its operating temperature range from −40 °C to 80 °C. This platform demonstrates excellent stability over more than 30 days and after 5000 cyclic tests, providing a reliable solution for extreme outdoor sports such as ice and snow events.^93^

**Fig. 5:**
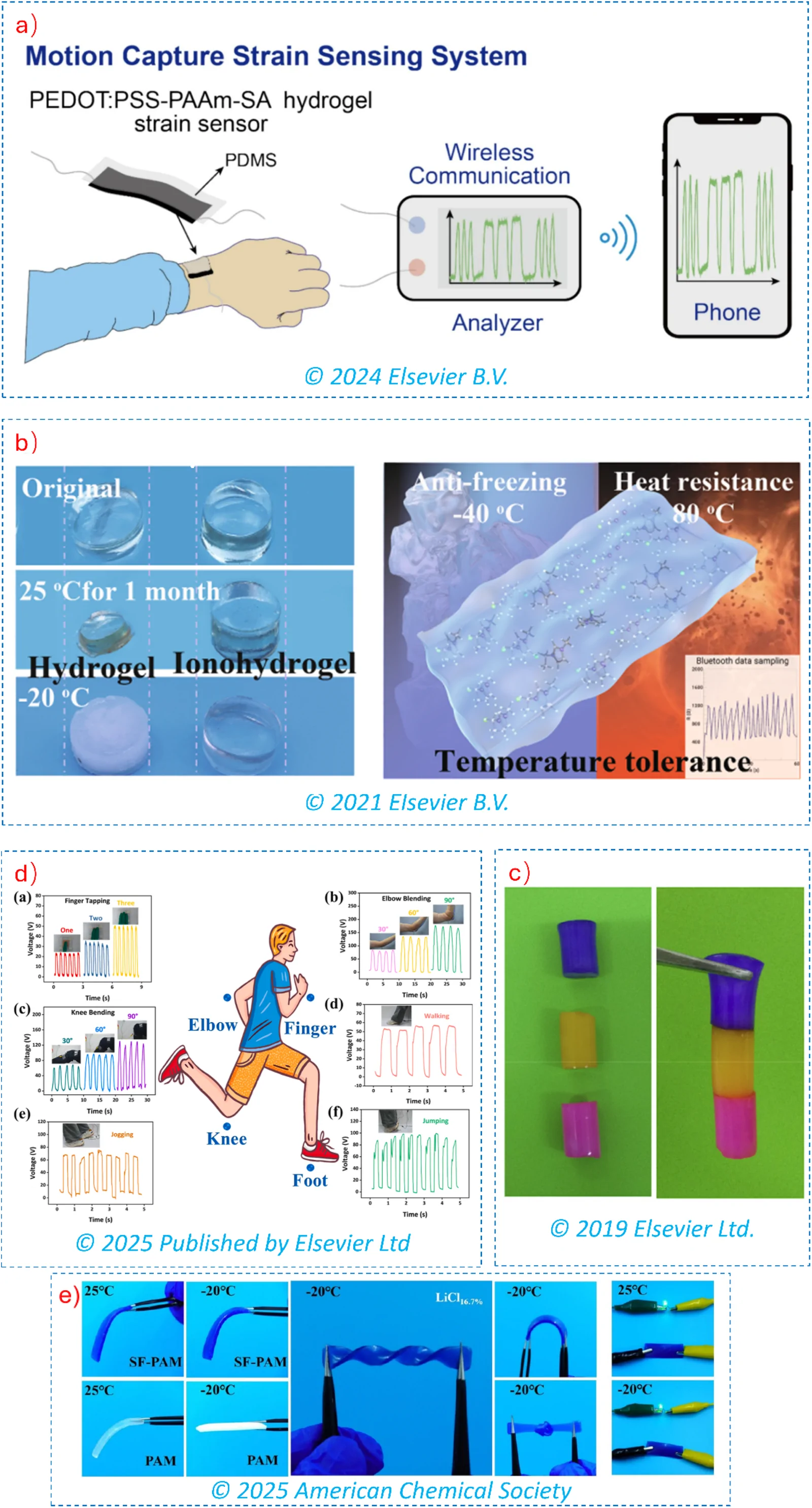
Application of advanced conductive hydrogel platforms in precise motion tracking and biomechanical energy harvesting. (a) Wireless motion capture system based on PEDOT : PSS/PAAm-SA hydrogels, demonstrating an ultra-low hysteresis of 1.52% at 500% strain. Reproduced from ref. 92 with permission from Elsevier,^92^ copyright 2024. (b) Highly transparent, hydrated ionic liquid-based hydrogels for all-weather monitoring across a wide temperature window (−40 °C to 80 °C). Reproduced from ref. 93 with permission from Elsevier,^93^ copyright 2021. (c) Self-powered landing impact monitoring utilizing PLM-TENGs with ultrahigh stretchability (1024%) and peak voltage output (594 V). Reproduced from ref. 94 with permission from Elsevier,^94^ copyright 2025. (d) Ionic strain/tactile sensors based on PAAm/PEO/LiCl exhibiting extreme compressive strength (556.58 MPa) for human activity detection. Reproduced from ref. 95 with permission from Elsevier,^95^ copyright 2019. (e) Stretchable, anti-freezing (−70 °C), and highly water-retentive (90% after 30 days) SF-PAM hydrogels integrated into smart systems for wearable e-skins. Reproduced from ref. 96 with permission from the American Chemical Society,^96^ copyright 2025.

#### 4.1.2 Quantification of impact force and landing energy

Abnormal landing impact force is a major risk factor for lower-limb injuries such as ACL rupture. As shown in Fig. 5c, a PLM double-network hydrogel constructed using lysine nanofillers and MgCl_2_ cross-linking achieves a tensile stress of 1.81 MPa and an ultimate strain of 1024%. A triboelectric nanogenerator based on this material, namely PLM-TENG, can generate a peak voltage of 594.0 V and a power density of 19.5 W m^−2^ without an external power supply. This platform has been successfully applied to gait recognition and high-impact landing monitoring and remains stable after 15 000 cycles.^94^ Similarly, as shown in Fig. 5d, an ionically conductive hydrogel based on PAAm/PEO/LiCl exhibits an impressive compressive strength of 556.58 MPa, enabling highly sensitive capture of ground reaction force distribution during high-jump sports such as basketball and volleyball. These data provide robust support for the design of sports footwear, clothing, and protective equipment.^95^

### 4.2 Physiological signal and multimodal interconnected monitoring

#### 4.2.1 Wearable electronic skin and fatigue feedback

Early detection of muscle fatigue is crucial for preventing compensatory injuries. As shown in Fig. 5e, a composite ionic hydrogel synthesized from silk fibroin and polyacrylamide (SF-PAM) not only exhibits an ultrahigh elongation of 1663%, but also maintains a water-retention rate as high as 90% after 30 days and demonstrates anti-freezing capability at −70 °C. Researchers have successfully integrated this hydrogel into a wearable electronic skin system and connected it with intelligent carts and gloves. This system can continuously provide feedback on muscle and joint status during exercise and offers a precise human–machine interaction interface for clinical rehabilitation assessment.^96^

#### 4.2.2 Remote monitoring and integration with 5G IoT

Modern sports load management is moving toward cloud-based monitoring. As shown in Fig. 6a, a carbon-based conductive hydrogel (TA/CNC/PVA/MWCNT-COOH) fabricated using direct ink writing (DIW) 3D printing technology achieves an ultrafast self-recovery rate of 97% and excellent conductivity of 0.47 S m^−1^ through a multiple hydrogen-bonding system. This system can not only harvest motion energy through a triboelectric nanogenerator (TENG), but also be innovatively integrated with the IoT and 5G technology. Such integration effectively overcomes spatial limitations and enables coaching terminals to remotely track athletes' physiological functions and load status in real time.^97^

**Fig. 6:**
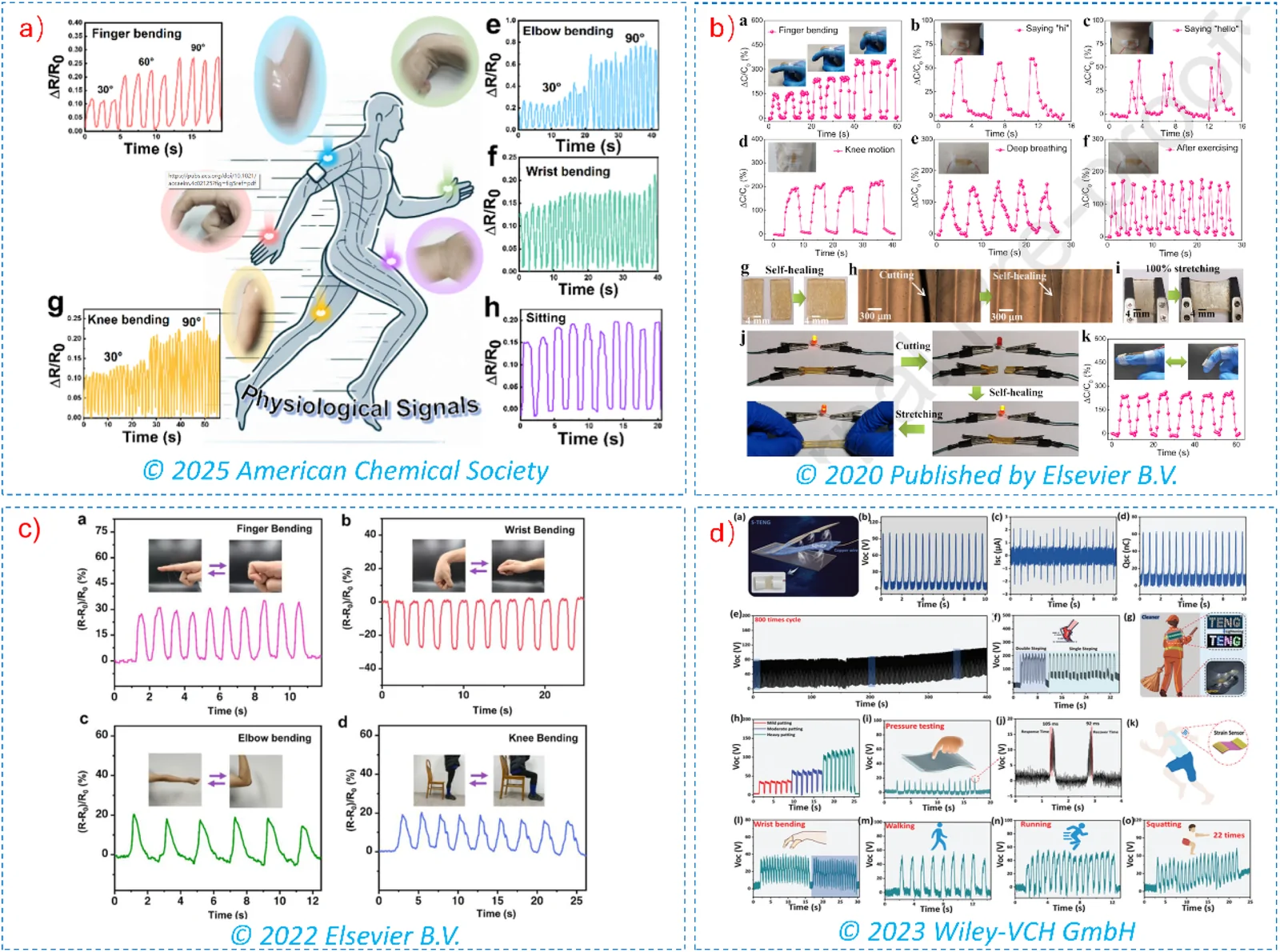
Innovative paradigms of conductive hydrogels in IoT integration, injury prevention, and smart rehabilitation. (a) DIW-printed self-healing hydrogels integrated with IoT and 5G technology for remote, real-time sports monitoring. Reproduced from ref. 97 with permission from the American Chemical Society,^97^ copyright 2025. (b) SPAH-based pressure sensors with programmable wrinkled surfaces (SCR strategy) enabling an ultra-broad sensing range (25–20 000 Pa). Reproduced from ref. 98 with permission from Elsevier,^98^ copyright 2021. (c) Wet-adhesive SF/TA@PPy hydrogels for reliable underwater motion perception and Morse code transmission. Reproduced from ref. 99 with permission from Elsevier,^99^ copyright 2022. (d) Multifunctional organohydrogel e-skins reinforced by a 3D goat skin fiber network (S@HCP) for UV-shielding, antibacterial, and self-powered rehabilitation monitoring. Reproduced from ref. 100 with permission from John Wiley and Sons,^100^ copyright 2023.

### 4.3 Sports injury prevention and intelligent rehabilitation

#### 4.3.1 Omnidirectional pressure sensing and early warning in complex environments

Multidimensional pressure sensing is fundamental for predicting potential sports injuries. As shown in Fig. 6b, a surface-wrinkled SPAH hydrogel fabricated using a stretching/competitive coordination (SCR) strategy achieves an extremely wide pressure response range from 25 Pa to 20 000 Pa and a high sensitivity of 3.19 kPa^−1^. It can comprehensively capture subtle respiratory signals as well as intense joint flexion and extension, providing multidimensional parameters for omnidirectional exercise-load warning.^98^ In addition, for special underwater sports such as swimming, as shown in Fig. 6c, a conductive hydrogel with strong wet adhesion can not only self-heal without external stimulation, but also maintain stable electrical output in underwater environments. It can even transmit Morse code by controlling motion changes, thereby filling the technological gap in real-time underwater posture monitoring.^99^

#### 4.3.2 Biomimetic multifunctional rehabilitation electronic skin

The evolution of postoperative rehabilitation devices toward high-dimensional biomimetic systems represents an inevitable future trend. As shown in Fig. 6d, through a “top-down” strategy, researchers integrated acrylic acid monomers with the three-dimensional fibrous network of natural goatskin and further loaded the system with Ag nanoparticles. This revolutionary multifunctional organic hydrogel electronic skin not only overcomes the poor mechanical properties of conventional hydrogels, but also endows the material with ultraviolet-shielding, strong antibacterial, and self-powered motion-monitoring capabilities. This green biomimetic platform based on the reuse of discarded animal “dead skin” provides a high-level materials-based solution for home-based postoperative rehabilitation and daily sports safety protection.^100^

Overall, the application evolution of conductive hydrogels in sports monitoring has successfully moved beyond the primary stage of single-dimensional passive data acquisition and entered a deeper phase characterized by multimodal active early warning, self-powered sensing, and profound Internet of Things interconnection. From overcoming a tiny dynamic hysteresis of 1.52%, to harvesting landing energy with an output voltage as high as 594 V, and further to breaking monitoring limitations in underwater and extremely cold environments down to −70 °C through 5 G and machine learning, conductive hydrogels have demonstrated irreplaceable advantages in flexible wearable systems. The ultimate vision of this field is to leverage such biomimetic and multifunctionally integrated flexible materials, deeply integrate them with edge computing and generative AI, and establish a digital physical-performance and health-management closed loop spanning all-weather scientific training monitoring, ultra-early intelligent injury warning, and boundaryless home-based rehabilitation. This vision is expected to fundamentally reshape the technological ecosystem of modern sports medicine.

## 5. Technical challenges and existing issues

Despite the rapid laboratory progress, several major challenges still hinder the translation of conductive hydrogel sensors from academic prototypes into commercial sports monitoring products.^101^ First, long-term durability remains inadequate: most hydrogels suffer from progressive performance degradation under repeated mechanical loading and continuous sweat exposure, limiting their service life far below commercial requirements. Second, batch-to-batch reproducibility is poor, and current fabrication methods (*e.g.*, one-pot synthesis or freeze–thaw cycling) are difficult to scale into continuous, low-cost manufacturing processes such as roll-to-roll coating or automated 3D printing. Third, there is a lack of standardized testing protocols and performance benchmarks specifically designed for soft hydrogel sensors under dynamic sports conditions, which creates uncertainty for industrial adoption and regulatory approval. Fourth, the seamless integration of the hydrogel sensing layer with flexible power sources, wireless communication modules, and edge-computing chips into a single, comfortable, and robust wearable platform is still technically immature. Finally, biocompatibility validation and medical-device certification pathways remain underdeveloped for most hydrogel systems. Addressing these interconnected barriers is essential for moving conductive hydrogel technology beyond the laboratory and into practical athletic and rehabilitation applications.

As shown in Fig. 7, the current limitations of this field are systematically reflected in four dimensions: intrinsic material defects, signal acquisition bottlenecks, difficulties in scalable manufacturing, and barriers to commercialization.

**Fig. 7:**
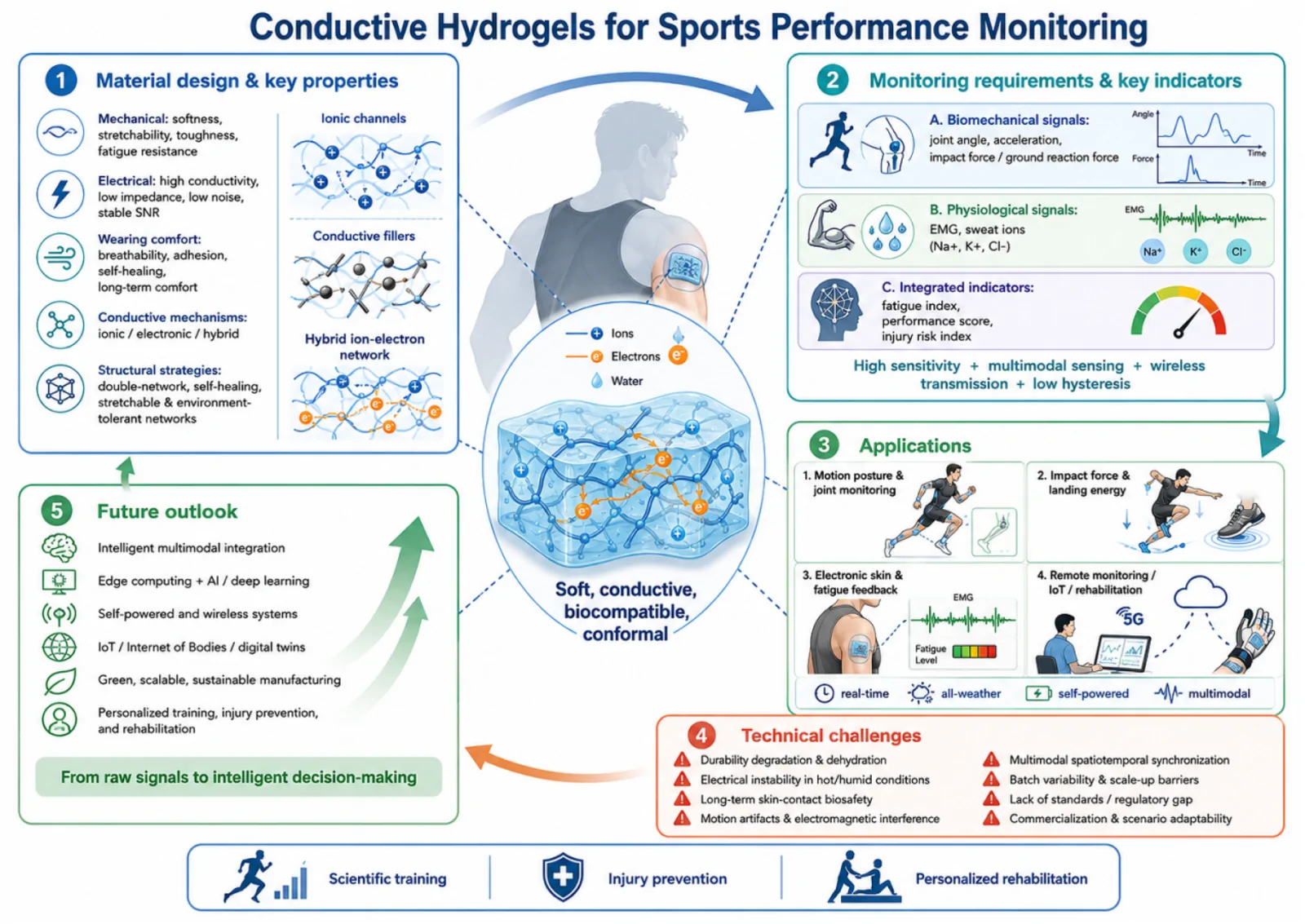
Current bottlenecks and the “Valley of Death” in translating conductive hydrogels from laboratory research to commercial sports monitoring applications. The successful commercialization requires overcoming critical challenges across four dimensions: intrinsic material limitations (*e.g.*, dehydration, bio-toxicity), signal acquisition barriers (*e.g.*, motion artifacts, multi-modal asynchrony), scalable manufacturing dilemmas, and the ultimate clinical/field translational gap.

### 5.1 Intrinsic material-level limitations

#### 5.1.1 Durability degradation and environmental vulnerability

The high water content of hydrogels is the basis for their softness and biocompatibility, but it also represents a critical weakness. Under long-term and high-intensity exercise exposure, these materials are prone to structural fatigue and performance degradation.^102^ Especially in hot and highly dynamic environments, water evaporation and ion dissipation can cause rapid dehydration and embrittlement of the gel matrix, leading to attenuated strain responses and irreversible signal drift.^103^ In addition, hydrogels containing natural components such as polysaccharides and silk fibroin are highly susceptible to microbial attack and degradation under high-temperature and high-humidity conditions. Therefore, how to substantially improve long-term structural stability under harsh service environments while maintaining biodegradability and environmental friendliness remains a fundamental contradiction in material design.^104^

A critical yet insufficiently addressed issue is the long-term stability of conductive hydrogels under repeated sweating conditions. During prolonged or high-intensity exercise, continuous exposure to sweat leads to several interrelated degradation pathways: (i) leaching of mobile ions that reduces ionic conductivity; (ii) excessive swelling or plasticization of the polymer network that compromises mechanical integrity and interfacial adhesion; and (iii) progressive accumulation of residual salts and metabolites that can cause signal drift or electrode-skin impedance increase. Although recent designs have introduced anti-swelling double networks, hydrophobic association, deep eutectic solvents, and dynamic self-healing bonds to alleviate these problems, most studies still report performance only under short-term or single-session sweating tests. Systematic evaluation of conductivity retention, adhesion strength, and sensing fidelity after multiple hours or consecutive days of sweat exposure remains rare. Establishing standardized cyclic sweating and accelerated aging protocols will therefore be essential for assessing the real-world durability of hydrogel sensors in sports applications.^102,104^

It should be noted that most conductive hydrogel sensors reported to date remain at the laboratory prototype stage (relatively low technology readiness levels), and systematic on-athlete validation under real training or competition conditions is still scarce.^70,91^ Different sports further impose distinct material requirements—for example, high impact resistance for landing-intensive sports, strong anti-swelling capability for aquatic sports, and reliable anti-freezing performance for winter sports—which have not yet been systematically matched to specific hydrogel designs.^35,66,70^

#### 5.1.2 Electrical instability in extremely humid and hot environments

Heavy sweating and exposure to external rainwater can drastically alter the osmotic pressure and ion concentration distribution inside hydrogels. Salts, lactate, and other metabolites in sweat can readily undergo side reactions with conductive components, disrupting the original conductive pathways and inducing severe baseline drift and irreversible performance loss.^105^ Although some studies have attempted to introduce hydrophobic coatings or semipermeable membranes, an unresolved trade-off remains between maintaining breathability and preserving skin-electrode conductive coupling. Achieving electrical stability in all-weather, high-humidity environments is still a severe challenge.^106^

#### 5.1.3 Long-term skin-contact biosafety

High-frequency and long-duration monitoring in competitive sports and postoperative rehabilitation requires sensors to coexist with sweat-soaked and vulnerable skin for extended periods, which can easily induce contact dermatitis or allergic reactions.^107^ More subtly, synthetic conductive polymers, metallic nanomaterials such as AgNWs, or carbon-based fillers introduced to achieve extremely high conductivity may pose potential risks of local irritation or even systemic toxicity if they detach from the matrix after wear and penetrate through pores. Therefore, a standardized full-lifecycle toxicological evaluation system for emerging conductive hydrogels is urgently needed.^108^

### 5.2 Bottlenecks in signal acquisition and processing

#### 5.2.1 Dynamic artifacts and multisource interference

Intense three-dimensional deformation during high-intensity activities such as sprinting and jumping can induce microscopic slippage at the sensor–skin interface, and the resulting motion artifacts may completely overwhelm weak physiological bioelectrical signals.^109,110^ Meanwhile, dense electronic devices in sports venues can introduce strong electromagnetic interference.^111^ Although front-end circuit shielding and back-end deep-learning denoising algorithms can partially alleviate this problem, achieving interference resistance at the material level, namely hardware-level signal-to-noise ratio enhancement, remains a technological blind spot.^112^

#### 5.2.2 Multimodal spatiotemporal synchronization

Multimodal sensing systems based on conductive hydrogels, while highly attractive for comprehensive sports monitoring, currently face several key limitations. First, different sensing modalities (mechanical strain, surface electromyography, and sweat biochemical signals) often exhibit mismatched response times, sensitivity ranges, and signal amplitudes, leading to spatiotemporal desynchronization that complicates real-time data fusion.^85^ Second, signal crosstalk between ionic, electronic, and electrochemical pathways within the same hydrogel network can degrade the fidelity of individual signals, especially under large deformation and continuous sweating. Third, the physical integration of multiple functional layers or regions into a single soft device increases structural complexity, interfacial impedance mismatch, and packaging challenges.^86^ Finally, the simultaneous acquisition and wireless transmission of multimodal data raise power-consumption and edge-computing demands that most current hydrogel platforms are not yet optimized to meet. Overcoming these limitations will require advances in asymmetric device architecture, adaptive signal-processing algorithms, and low-power system-level integration.

Integrated sensing of multimodal signals, including mechanical strain, EMG, and sweat biomarkers, represents a future direction.^113^ However, different signal sources inherently differ in sampling frequency and response latency. In highly dynamic scenarios such as ACL injury warning, millisecond-level phase mismatch between impact force and EMG signals may directly invalidate predictive models.^114^ Current strategies that rely on complex back-end data fusion substantially increase the power consumption and volume of wearable devices. Therefore, developing highly integrated and low-power physical synchronization architectures at the hardware level is essential for achieving wireless and imperceptible monitoring.^115^

### 5.3 Difficulties in scalable manufacturing and standardization

A further practical obstacle is the limited reproducibility and batch-to-batch consistency of current conductive hydrogel fabrication methods. Laboratory procedures such as one-pot synthesis, freeze–thaw cycling, or manual solution casting are often highly sensitive to minor variations in monomer/filler concentration, cross-linking time, temperature, and ambient humidity. Consequently, hydrogels prepared in different batches or laboratories can exhibit substantial differences in conductivity, modulus, adhesion, and sensing metrics, which complicates performance benchmarking and quality control. The absence of standardized fabrication protocols and in-process quality metrics exacerbates this problem. Transitioning to automated, continuous manufacturing techniques (*e.g.*, roll-to-roll coating or direct ink writing 3D printing) together with the development of more robust, formulation-tolerant chemistries will be essential to achieve the batch consistency required for commercial sports monitoring products.^45,65^

#### 5.3.1 Batch variability and barriers to process scale-up

Most high-performance conductive hydrogels currently rely on highly stringent and complicated laboratory fabrication processes, such as freeze–thaw cycling and *in situ* ultraviolet polymerization. These discontinuous processes are difficult to adapt to roll-to-roll large-scale industrial manufacturing, resulting in high production costs and considerable batch-to-batch fluctuations in electromechanical properties.^116^ The lack of consistency in large-scale fabrication prevents these advanced materials from entering true commercial production lines.^117^

#### 5.3.2 Lack of industry standards and regulatory frameworks

As core sensing materials that are directly attached to the human body, conductive hydrogels currently lack dedicated testing standards for medical devices or sports equipment.^118^ Existing approval and testing procedures often borrow standards from conventional medical gels or silicone electrodes, which cannot fully cover hydrogel-specific indicators such as dynamic electrical performance, moisture permeability, and long-term fatigue resistance. This severe absence of standards has greatly slowed their translational approval process for clinical rehabilitation and competitive sports applications.^119^

### 5.4 Barriers to commercial translation

Critical barrier to translation is the absence of standardized evaluation protocols specifically tailored for conductive hydrogel sensors in sports applications. Current literature reports sensing performance under highly variable conditions—different strain amplitudes and frequencies, humidity levels, electrode geometries, and signal-processing methods—which severely limits cross-study comparison and industrial benchmarking.^85^ For sports monitoring, key metrics that urgently require standardization include: (i) dynamic electrical response and hysteresis under high-frequency cyclic loading that mimics gait or jumping; (ii) conductivity and adhesion retention after prolonged or repeated sweat exposure; (iii) skin-electrode contact impedance under large skin deformation; (iv) motion-artifact rejection capability during intense activity; and (v) multi-day operational durability under realistic temperature and humidity fluctuations. Establishing hydrogel-specific testing standards, potentially extending existing ISO and ASTM frameworks for flexible electronics and medical electrodes, will be essential for accelerating regulatory approval and commercial adoption.^85^ In addition, estimated production costs remain largely unreported, and clear pathways for medical-device certification are still lacking, further hindering industrial translation.

#### 5.4.1 Scenario generalization and adaptability gap

Impressive data obtained under laboratory conditions are often substantially weakened when confronted with abrupt environmental changes and interindividual variability in real sports settings.^120,121^ Different sports impose highly distinct requirements on sensors. High-contact ball sports demand impact-resistant flexibility, whereas endurance running emphasizes breathability, sweat management, and long battery life. A single material formulation is unlikely to achieve generalized adaptation across all sports scenarios.^122,123^

#### 5.4.2 Industrial design and cost disadvantages

A further systems-level challenge lies in the integration of power sources, wireless communication modules, and hydrogel sensing layers into a single, comfortable wearable platform. Soft hydrogels exhibit tissue-like compliance, whereas most current batteries, energy harvesters, and wireless chips remain relatively rigid, leading to mechanical mismatch, interfacial stress concentration, and potential delamination under large deformation.^86^ Taining stable electrical interconnects that withstand repeated stretching and continuous sweat exposure is also difficult. In addition, the limited energy density of flexible power sources and the power demands of continuous multimodal sensing plus wireless transmission constrain operational lifetime. These integration issues currently prevent most laboratory hydrogel sensors from evolving into fully self-contained, long-term wearable systems suitable for real-world sports use.

Most research-grade hydrogel sensors remain at a rudimentary stage characterized by exposed chips and wired connections, far from the imperceptible encapsulation, aesthetically pleasing user-interface design, and highly integrated wireless modules required for consumer products.^124,125^ High material synthesis costs and fragile back-end packaging make these devices commercially less competitive than existing mature rigid wearable devices, such as smartwatches. Overcoming this barrier requires deep interdisciplinary integration among materials science, microelectronics, and industrial design.^126^

Overall, the multiple technical barriers faced by conductive hydrogels indicate that this field is currently undergoing a painful transition from a “materials game” focused on stacking extreme parameters toward system-level engineering implementation. To cross the “valley of death” from the laboratory to commercial sports applications, future breakthroughs cannot rely solely on the repair of individual technical points. At the intrinsic material level, multiphysics simulations should be used to guide the targeted design of water-retaining and disturbance-resistant structures. At the signal level, multimodal temporal discrepancies must be eliminated through deep coupling between edge computing and low-level hardware architectures. At the industrial level, the establishment of rigorous dynamic testing standards and the development of continuous large-scale fabrication processes will be decisive. Only by tightly integrating material innovation with engineering implementation can conductive hydrogels truly reshape the technological foundation of next-generation intelligent sports equipment.

Before conductive hydrogels can achieve widespread adoption in sports medicine and athlete monitoring, several critical knowledge gaps must be addressed. These include: the limited long-term durability of hydrogels under combined mechanical cycling and continuous sweat exposure; the absence of standardized testing protocols that enable fair comparison with commercial sensors; poor batch-to-batch consistency of current fabrication methods; insufficient long-term biocompatibility and safety data under real wearing conditions; challenges in achieving reliable multimodal signal synchronization and low-latency on-device data fusion; and the lack of clear regulatory pathways for medical-device certification. Closing these gaps through coordinated advances in materials design, standardized evaluation, scalable manufacturing, and system-level integration will be decisive for the practical translation of this promising technology.

## 6. Future Perspectives

With the deep convergence of materials science, flexible electronics, and generative AI, the application of conductive hydrogels in sports performance monitoring is entering a transformative stage. Future developments are expected to break through the limitations of single-signal acquisition and evolve comprehensively toward multimodal integration, intelligent analysis, and large-scale industrial ecosystems.^127^ As illustrated in Fig. 8, the key pathways for overcoming current technical bottlenecks and leading the next generation of intelligent wearable devices can be summarized in the following five dimensions.

**Fig. 8:**
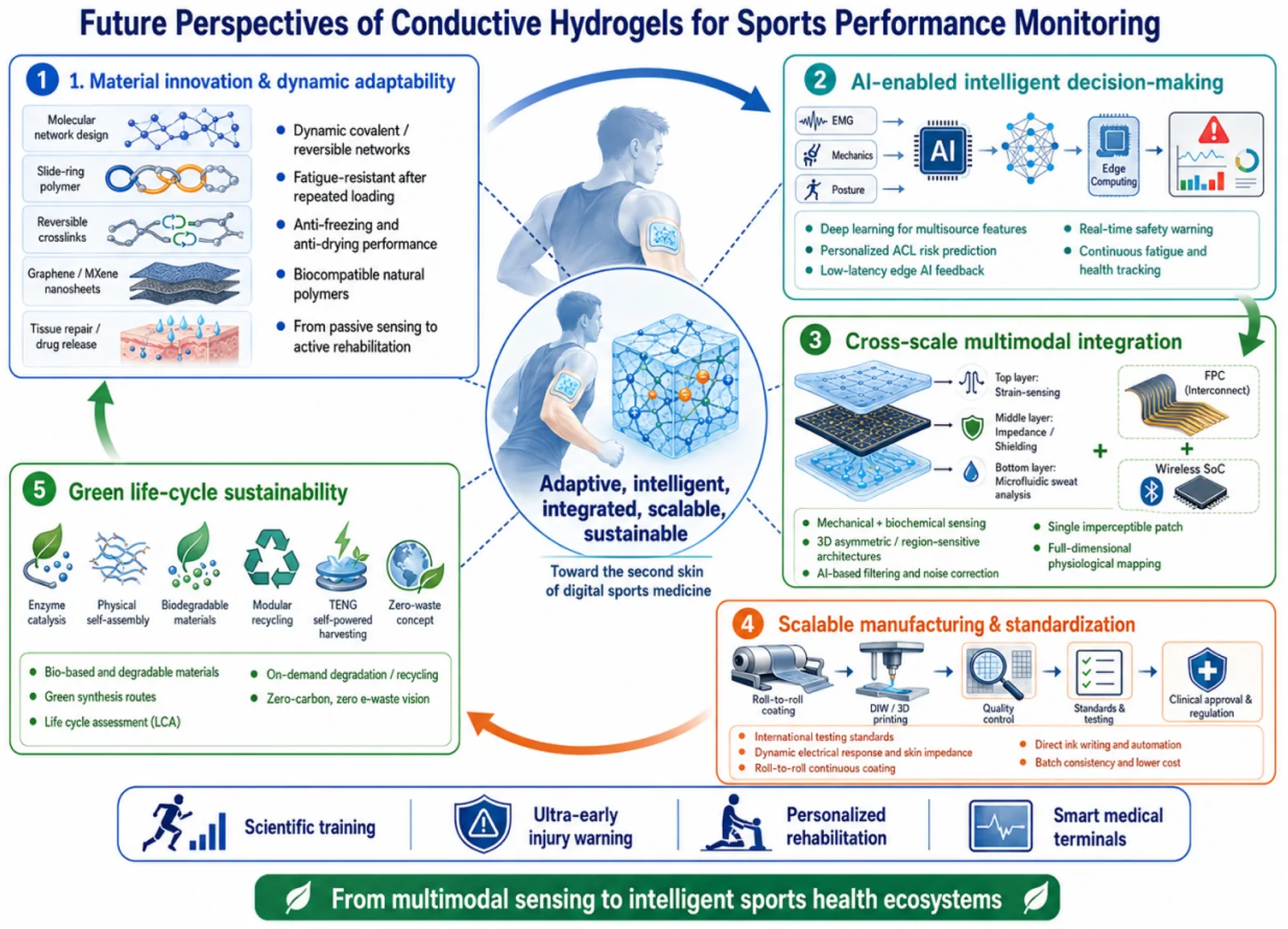
Future perspectives of conductive hydrogels for sports performance monitoring.

### 6.1 Material innovation and dynamic adaptive performance

Breaking through the limits of static performance and endowing materials with dynamic environmental adaptability is a key direction for fundamental innovation.^128^ To address the current shortcomings of water loss and fatigue-induced degradation, future research should focus on molecular-level network design, such as the incorporation of slide-ring polymers, multiple reversible physical crosslinks, and dynamic covalent bonds, so that the material can maintain structural integrity even after millions of high-frequency loading cycles.^129^ At the same time, three-dimensional percolation networks can be constructed by incorporating two-dimensional nanomaterials such as graphene and MXene, thereby imparting anti-freezing and anti-drying capabilities to hydrogels for all-weather outdoor sports monitoring.^130^ In addition, biosafety and multifunctional integration will become essential requirements. Replacing highly toxic matrices with natural polymers such as silk fibroin and alginate,^131^ while integrating tissue repair and controlled drug release functions, will enable conductive hydrogels to evolve from passive monitoring platforms into active rehabilitation intervention systems.

Among future material-design strategies, several approaches appear particularly promising for simultaneously improving durability, anti-dehydration behavior, and environmental tolerance. First, replacing conventional water with deep eutectic solvents (DESs) or other low-volatility solvents can fundamentally suppress dehydration and extend operational lifetime under dry or high-temperature conditions.^41,56^ Second, the rational design of multi-level dynamic networks—combining hydrogen bonding, hydrophobic association, and metal–ligand coordination—offers a pathway to high fatigue resistance and rapid self-healing under the repeated large-strain cycles typical of sports activities.^65^ Third, the integration of three-dimensional continuous conductive skeletons (*e.g.*, graphene or MXene frameworks) with phase-change or lyophilic components enables both mechanical robustness and active thermal/humidity management, as demonstrated by systems that remain functional at −25 °C with excellent water retention.^66^ Finally, the increasing use of green multifunctional building blocks such as lignin and natural polyphenols provides a sustainable route to combine conductivity with inherent UV-shielding, antioxidant, and anti-dehydration properties.^61,120^ Collectively, these strategies are expected to move conductive hydrogels from laboratory prototypes toward reliable, all-weather sports monitoring devices.

### 6.2 Intelligence and deep integration with AI

Driven by the rapid development of the Internet of Things and edge computing, sports monitoring is progressing from simple data recording toward intelligent decision-making.^132^ On the data-analysis side, deep learning algorithms can extract multisource features from high-dimensional EMG, mechanical, and motion-posture data to establish highly accurate personalized prediction models for ACL injury risk.^133^ On the feedback side, embedding lightweight AI models into flexible sensing devices can enable real-time motion assessment and safety warning with extremely low latency, thereby substantially reducing the incidence of acute injuries.^134^ This synergistic “AI + hydrogel” architecture will continuously characterize athletes' fatigue and health trajectories, allowing conductive hydrogels to become fully integrated into the broader ecosystem of intelligent sports.^135^

The integration of artificial intelligence (AI) and advanced data-fusion strategies is becoming indispensable for unlocking the full potential of conductive hydrogel sensors in sports performance monitoring. Hydrogel platforms can simultaneously generate high-dimensional signals (strain, pressure, sEMG, sweat ions, *etc.*), yet the raw data are often noisy, asynchronous, and difficult to interpret in real time. Recent studies have demonstrated that lightweight deep-learning models, such as two-dimensional convolutional neural networks (2D-CNNs), can achieve high recognition accuracy (up to 99.37%) for complex motion patterns even under challenging underwater conditions.^70^ Machine-learning-based fusion of biomechanical and physiological features has also been used to estimate fatigue indices and injury risk more reliably than single-modality approaches.^13,78,80^

Looking forward, the key technical challenges lie in developing edge-compatible AI algorithms that can run on resource-constrained wearable hardware with low latency, as well as robust data-fusion frameworks capable of handling the spatiotemporal misalignment and crosstalk inherent to soft multimodal hydrogel sensors.^134,135^ The combination of on-device AI with hydrogel sensing is expected to transform passive data-collection patches into active, intelligent decision-making systems that provide real-time feedback for training optimization and injury prevention.

### 6.3 Cross-scale multimodal integrated systems

Future monitoring platforms must achieve the simultaneous capture of mechanical strain and physiological/metabolic information, such as sweat ions, within highly limited physical space.^136^ To address the spatiotemporal decoupling of multimodal signals, future hardware should evolve toward three-dimensional asymmetric and region-sensitive architectures: the top layer for deformation sensing, the bottom layer for microfluidic sweat collection, and the middle layer for impedance matching and signal shielding. In addition, through the integration of highly compact flexible printed circuits (FPCs) and miniaturized wireless system-on-chip (SoC) devices,^137^ together with AI-driven adaptive filtering and noise-correction algorithms, the ultimate goal of a single imperceptible patch for full-dimensional physiological mapping can be realized, thereby empowering intelligent rehabilitation robots and smart medical terminals.^138^

### 6.4 Large-scale manufacturing and the establishment of standardization systems

To cross the “valley of death” from laboratory concept to commercial product, it is urgent to establish dual drivers of standardization and industrialization. First, internationally recognized testing standards specifically for flexible conductive hydrogels must be developed, covering hydrogel-specific indicators such as dynamic electrical response, long-term biocompatibility, and skin–electrode interfacial impedance, so as to clear the way for clinical approval and commercialization.^139^ Second, in terms of manufacturing, inefficient traditional methods such as freeze–thaw cycling must be replaced by automated and green fabrication technologies, including roll-to-roll continuous coating and direct ink writing 3D printing, in order to fundamentally solve the industrial bottlenecks of poor batch-to-batch consistency and high production cost.^140^

### 6.5 Green and sustainable development across the entire life cycle

Under the global “dual-carbon” strategy, the green sustainability of conductive hydrogels will become a core technological barrier. Future research should completely move away from toxic chemical cross linkers and instead adopt green synthetic routes such as enzyme catalysis and physical self-assembly, with priority given to natural, bio-based, and biodegradable materials.^141^ More importantly, material design should be extended to life cycle assessment (LCA) and full product life cycle management. Through modular packaging, on-demand degradation or recycling mechanisms, and self-powered sensing based on human-motion energy harvesting, such as integrated triboelectric nanogenerator (TENG) technology, it may be possible to develop truly green intelligent sports monitoring equipment with zero carbon emissions and zero electronic waste.^142^

In summary, the evolution of conductive hydrogels in sports monitoring represents a systematic revolution spanning material synthesis, multidimensional signal sensing, high-level AI algorithms, and industrial-scale manufacturing. The future of this field requires not only local breakthroughs in individual material properties, but also deep integration across materials science, microelectronic engineering, sports biomechanics, and computer science. Only by ensuring stability under extreme environments, overcoming the barriers of multimodal spatiotemporal coupling, and fully establishing scalable and sustainable industrial chains can conductive hydrogels truly evolve into the “second skin” of next-generation digital humans, providing indispensable support for scientific athletic training, ultra-early injury warning, and personalized medical rehabilitation.

## 7. Conclusion

Looking ahead, owing to their unparalleled intrinsic flexibility and multimodal sensing advantages, conductive hydrogels are expected to evolve into a core technological foundation for the integration of next-generation intelligent sports equipment and flexible electronics. With their deep convergence with 5G IoT, advanced edge computing, and generative AI, future flexible sensing platforms will fundamentally overcome the data isolation and spatiotemporal limitations imposed by conventional rigid devices. This deep hardware–software synergy will drive sports monitoring to evolve from passive single-signal acquisition terminals into active intelligent decision-making hubs capable of autonomous learning and real-time feedback.

This technological leap will not only establish full-dimensional digital mapping between microscopic physiological metabolism and macroscopic biomechanics, but also enable a seamless health-management closed loop spanning all-weather scientific training guidance, ultra-early warning of high-risk injuries, and personalized precision medical rehabilitation. Ultimately, the comprehensive breakthrough and industrial translation of conductive hydrogel technology will irreversibly reshape the ecological boundaries of modern sports medicine, human performance science, and competitive sports, leading intelligent wearable devices into a new era of deep human-machine symbiosis and ubiquitous data interconnection.

## Ethics statement

This article is a review and does not involve any studies with human participants or animals performed by the authors. Therefore, ethical approval and informed consent are not required.

## Author contributions

Jiahang Liu and Xiuya Zhang contributed equally to this work. Jiahang Liu and Xiuya Zhang were responsible for the literature collection, data analysis, and manuscript drafting. Shijun Liu supervised the study, contributed to the conceptualization and critical revision of the manuscript, and provided overall guidance for the research. All authors read and approved the final manuscript.

## Conflicts of interest

The authors declare that they have no competing interests.

## Data Availability

Data sharing is not applicable to this article as no new datasets were generated or analyzed during the current study. All figures included in this review are reproduced from previously published studies, and appropriate permissions and citations have been provided where required.
